# Development and Psychometric Evaluation of the Cardiac Rehabilitation Adherence Tool (CRAT)

**DOI:** 10.3390/jcdd6030025

**Published:** 2019-07-09

**Authors:** Behzad Hamedani, Hooman Shahsavari, Sara Amaniyan, Christina Sieloff, Mojtaba Vaismoradi

**Affiliations:** 1 School of Nursing and Midwifery, Tehran University of Medical Sciences, 1419733171 Tehran, Iran; 2 College of Nursing, Montana State University, Bozeman, MT 59717, USA; 3 Faculty of Nursing and Health Sciences, Nord University, 8049 Bodø, Norway

**Keywords:** cardiac rehabilitation, heart disease, health care evaluation mechanisms, treatment adherence and compliance, psychometrics properties

## Abstract

Patients with cardiac diseases can achieve the greatest benefit from cardiac rehabilitation through modification of their unhealthy behaviors. This study aimed to develop and examine the psychometric properties of the Cardiac Rehabilitation Adherence Tool (CRAT), which was designed to assess patients’ adherence to cardiac rehabilitation. In this instrument development study, the items of the CRAT were extracted through a comprehensive literature review. The CRAT was assessed in terms of validity and reliability. Exploratory factor analysis was conducted to assess its construct validity, which led to the development of a tool containing 57 items and five dimensions including “acceptance of the rehabilitation center”, “being interested in health”, “feeling a need”, “personal control over the situation”, and “encouragement and advice.” These five factors accounted for 45.23% of the observed variance. The Cronbach’s alpha was 0.935. The test-retest method supported the stability of the instrument (*r* = 0.95). Health care professionals can use the CRAT to examine factors influencing the patient’s decision to leave cardiac rehabilitation and design strategies for improving their adherence to the rehabilitation program.

## 1. Introduction

Cardiovascular diseases (CVD) are the leading cause of death [1], chronic disability, loss of independence, and impaired quality of life across the globe [2]. CVD are responsible for 17.5 million deaths per year globally [3]. According to the report by the American Heart Association (AHA), based on the 2013 death rate, more than 2200 persons die of CVD each day with an average of one death every 40 s [4]. Therefore, the United Nations has adopted targets for reducing mortality related to CVD in terms of reduction of behavioral risk factors and a 25% reduction in premature mortalities through rehabilitation initiatives [5].

Attendance at a cardiac rehabilitation (CR) program has been shown to lead to a 26% reduction in cardiac mortalities over three years and a 31% reduction in cardiac re-hospitalizations over a 12-month period [6,7]. This program is considered a health behavior change intervention, that promotes healthy behaviors among patients with CVD [8]. It aims at preventing disease progression, promoting patients’ quality of life, and decreasing disease-related disabilities and mortalities [9].

Despite the benefits of participation in a CR program, patients’ adherence is often scarce. In a study in the USA, only 44% of eligible patients attended a CR program with 30–50% of them prematurely abandoning it [10]. Distance to the CR center, the cost of rehabilitation, individuals’ viewpoints, and patient’s perceptions about their diseases were associated with adherence or non-adherence to a CR program [9,11].

Given the importance of the CR program in recovery from CVD [12], a valid and reliable instrument is required to evaluate patients’ adherence to the program. Through the identification of features of adherence to the CR program using a specific tool, related barriers and facilitators can be identified in relation to the characteristics of cardiac patients, healthcare staff, severity of the illness and the healthcare system. Therefore, the present study aimed to develop and examine the psychometric properties of an instrument to assess patients’ adherence to CR and promote it through identifying factors influencing patients’ decision to leave the CR program.

## 2. Materials and Methods

The Research and Ethics Committee affiliated with Tehran University of Medical Sciences approved the study (decree code: 9111196056). Official permission to enroll cardiac patients was obtained from the referral CR centers. Patients were informed of the aim and method of the research, and the voluntary nature of participation in the study. They could withdraw from the study at any time without any effect on their rehabilitation process. The written consent form was signed by those patients who willingly agreed to participate.

### 2.1. Research Setting

Two large CR centers affiliated with two educational healthcare hospitals in Tehran were chosen for data collection. The cardiac patients were referred to the CR centers by hospitals or healthcare clinics. The CR centers provided various healthcare services to patients with CVD including individual and group counselling, physical examinations, education of heart-healthy living, exercise programs monitored by staff nurses, and ongoing and follow-up care. The duration of each CR session depended on patients’ general health status and needs.

### 2.2. Participants

This study was conducted between February 2015 and June 2016. The patients were selected using a convenience sampling method based on the following inclusion criteria: (1) suffering from CVD, (2) current participation in the CR program, (3) ability to read and write in Farsi, and (4) willingness to take part in the study. The researcher (BH) referred to the CR programs, assessed the patient list in terms of the eligibility criteria, and invited them to take part in the study.

### 2.3. Procedure

This instrument development study was structured in the stages of item generation, face validity, content validity, construct validity, and reliability:

#### 2.3.1. Item Generation: Analysis of Available Data and Literature Review

The findings of an in-depth qualitative study conducted in Iran by Shahsavari et al. [9] were used to develop an initial item pool. It used semi-structured interviews with cardiac patients referred to a large CR center in a teaching hospital in an urban area of Iran. They interviewed eight patients regarding their experiences of CR sessions and performed data analysis through a phenomenological approach. It led to the development of themes including “experience of heart event”, “life quality after event”, “the reason for compliance and attendance”, and “effective experiences of participation in CR.” The developed themes and codes under each theme were used for item generation in the current study.

**Figure 1:**
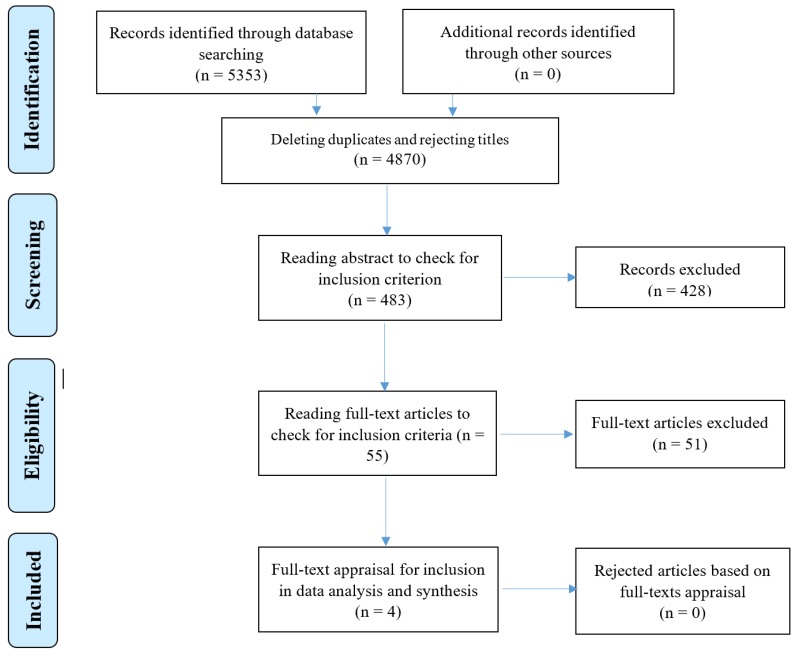
The progression of the search strategy according to the PRISMA.

In addition, a comprehensive literature review was performed to retrieve articles related to attendance and adherence to CR programs in online databases consisting of PubMed (including Medline), Scopus, SID, Magiran, and Iran Medex from 2000 to 2016. The keywords were used independently or in different combinations of appropriate Boolean operators such as (heart disease OR cardiovascular diseases OR cardiac rehabilitation) AND (adherence OR compliance OR commitment). The search yielded 5353 potentially relevant articles. The researchers assessed the titles, abstracts, and full-texts of articles to select the most relevant ones, based on the inclusion criteria of focus on adherence to CR, published in peer-reviewed journals, and being available online. Finally, 55 articles were selected and after full-text reading and appraisals, four relevant instrument development articles were chosen (Figure 1) and their findings were used for item generation (Table 1). Potential items were extracted from these studies and were incorporated into the item pool.

The initial instrument entitled the Cardiac Rehabilitation Adherence Tool (CRAT) with 109 items was created for psychometric testing.

#### 2.3.2. Face Validity

Face validity evaluated if the instrument measured the same topics for which it was designed [17]. Using pilot testing, the perspectives of 10 patients and 10 healthcare professionals were sought in terms of “the difficulty level of concepts”, “relevance”, “confusion”, and “misperceptions” of the items.

#### 2.3.3. Content Validity

Content validity was about how much the items of the instrument were representative of the study phenomenon and covered it [18]. For the content validity ratio (CVR), 10 experts in the fields of cardiac nursing and rehabilitation assessed each item based on a three-point Likert scale as “essential”, “useful but not essential”, and “not essential.” According to the Lawshe’s table [19], if the CVR was more than 62% based on the evaluation by the experts, the item was recognized as significant and remained in the instrument. The content validity index (CVI) was calculated based on the experts’ agreements on the relevance of each item. Each item was assessed in terms of “simplicity and fluency”, “relevance” and “clarity or transparency” based on a four-point Likert scale [20].

#### 2.3.4. Construct Validity

Factor analysis was used to measure the CRAT’s construct validity. There is little agreement among researchers on the number of samples required for conducting factor analysis. While a larger sample size has been suggested, realistically, the number of available subjects may restrict the goal. Accordingly, three samples per each item or at least 200 subjects were considered a fair sample size in this study [21,22]. Additionally, the Kaiser–Meyer–Olkin (KMO) and Bartlett’s test helped with confirming the adequacy and suitability of the samples for statistical analysis [21]. The method with a varimax rotation was used for extracting factors. The loading factor for each item in the rotated matrix should be at least 0.3 or higher [23]. In addition, an eigenvalue of less than 0.3 was considered the criterion for deleting items. Additionally, the scree plot chart helped with determining the number of factors. It showed the eigenvalues on the y-axis and the number of factors on the x-axis. The point of curve slope suggested the number of factors generated by the analysis [22].

#### 2.3.5. Reliability

The internal consistency (Cronbach’s alpha) and test-retest methods within a two-week interval were used. The minimum level of 0.7 was recommended for the Cronbach’s alpha coefficient [24]. For stability of the CRAT, the intraclass coefficient (ICC) was calculated for all dimensions [24]. Subsequently, in line with the suggestion of having a larger sample size to prevent measurement bias [18], 20 patients participating in the CR program completed the CRAT within a two-week interval and the minimum ICC value was considered 0.4 [25].

### 2.4. Data Analysis

All analyses were performed using the SPSS software version 16 for Windows (SPSS Inc., Chicago, IL, USA). Descriptive statistics including frequencies, percentages, means, and standard errors were used to illustrate the study population. The CVR was calculated based on the Lawshe’s table. Factor analysis, Cronbach’s alpha and test-retest, and the ICC were used to measure construct validity, internal consistency, and stability of all dimensions, respectively. *p* < 0.05 was considered statistically significant.

## 3. Results

### 3.1. Demographic Characteristics of the Patients

In this study, 210 subjects were recruited to participate. They were mostly (58%) male with an average age of 55 years (range 39–71 years). Additionally, 87% and 93% of them were married and had health insurance, respectively. Furthermore, 76% of the patients had a history of alcohol consumption, tobacco smoking, or substance abuse (based on a history of positive opioid laboratory test) as a leading cause of CVD. More details of the demographic characteristics of the patients is provided in Table 2.

### 3.2. Face Validity

Suggestions by patients and the experts led to minor modifications in the items. For instance, the word ‘patient’ was changed to “me” or “I”. The word ‘most often’ was removed from two questions. While it was suggested that negative items were changed to positive ones, no change was made in the items’ structures to disrupt a response set where the patients responded favorably or unfavorably to all items [26].

### 3.3. Content Validity

The initial CRAT had 109 items. However, suggestions by the expert panel led to merging 13 items and also deleting 25 items due to a CVR less than 62%. After merging and deleting items, the CRAT had 71 items and was formulated in the form of an instrument with a Likert scale (completely disagree = 1, disagree = 2, neutral = 3, agree = 4, completely agree = 5) for the field study. It is noted that some items were negative and had a reverse scoring.

### 3.4. Factor Analysis

**Figure 2:**
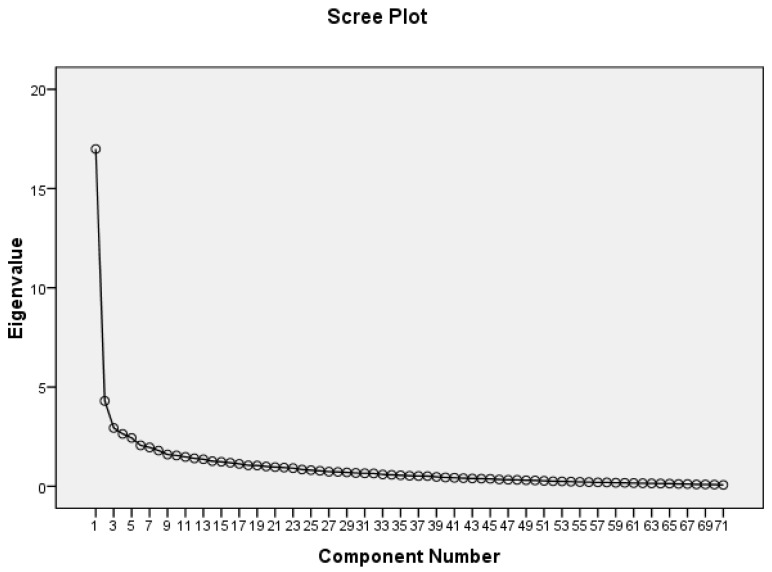
The scree plot of the items in exploratory factor analysis.

Exploratory factor analysis with a varimax rotation was conducted with data collected from a sample of 210 patients. The Bartlett’s test was statistically significant (*p* < 0.001). In addition, the value of the KMO was 0.82 indicating the sufficiency of the sample size for performing exploratory factor analysis [20]. To determine the number of factors, the eigenvalue of each item was used, and those items with a factor load less than 0.3 were excluded [22]. The scree plot chart also helped determine the number of factors related to adherence to the CR program. Therefore, five factors were identified to explain the structure of the CRAT and assess cardiac patients’ adherence to the CR program (Figure 2).

Varimax rotation was used in the next step of exploratory factor analysis. These five factors accounted for 45.23% of the observed variance. Nine items had a factor loading less than 0.3 and were excluded. Furthermore, five items were deleted because they had similar loading values and could not be placed in a special dimension (Table 3, see Supplementary Materials). The highest loading factor for each item was considered the criterion to classify it under each factor [23].

### 3.5. Reliability

The Cronbach’s alpha coefficient was reported 0.935, indicating an appropriate internal consistency of the whole CRAT. In addition, the Cronbach’s alpha coefficient for each dimension was reported as acceptable (Table 3). Although an alpha of at least 0.70 is considered desirable, a cut-off of 0.60 is most common in exploratory research [27,28]. Therefore, the reported Cronbach’s alpha for the last dimension (0.612) was considered acceptable in this study. Given that the overall ICC for the CRAT was 0.95 and for the dimensions varied from 0.91 to 0.94, stability of the CRAT was confirmed. Finally, the CRAT was established with 57 items and five dimensions of “the acceptance of the rehabilitation centers”, “being interested in health”, “feeling a need”, “personal control over the situation”, and “encouragement and advice.”

## 4. Discussion

The purpose of this study was to develop and examine the psychometric properties of the CRAT to assess patients’ adherence to the CR program. The CRAT aimed to understand the facilitators of and barriers to adherence to CR programs by cardiac patients. It was composed of 57 items and five dimensions. The dimensions and related contents of the CRAT were discussed below and put to an international perspective.

### 4.1. The Acceptance of the Rehabilitation Centers

This dimension aimed to investigate factors leading to the patients’ greater acceptability of the CR program by considering their perspectives and intentions. It is believed that intentions are the most important predictors of health behaviors [29]. When an individual develops an inclination toward a health behavior, the ‘good intention’ should be transformed to detailed instructions on how to perform the desired action. The intention needs to be supplemented by others [30]. The items of this dimension included happiness regarding the rehabilitation environment, facilities, communication between the patient and healthcare staff, and attention to patients’ wishes and needs. Similarly, Turk-Adawi et al. [31] stated that organizational factors such as adequate space and equipment, and patient satisfaction promoted patients’ commitment and adherence to the rehabilitation program. Additionally, Resurrección et al. [32] and Pesah et al. [33] believe that an appropriate attention to logistical reasons in terms of transport and distance to the rehabilitation center, staffing and equipment, and convenient rehabilitation timing is needed to prevent cardiac rehabilitation dropouts.

### 4.2. Being Interested in Health

This dimension addressed the patients’ understandings of the usefulness of the CR program and its impact on their attitudes of health. One required process for behavior change is the individual’s understanding, which is often some level of risk awareness including the feeling of vulnerability to cardiac events [34]. Education is a core component of a CR program that promotes patients’ understanding and adherence to secondary prevention strategies [14,15]. McCarthy et al. [35] reported that understanding the severity of the disease led to a better adherence to the recommended regimen. Reges et al. [36] found that the patient’s age, diagnosis at discharge, socioeconomic position, and perceived benefits of exercise influenced the patients’ participation in and adherence to the rehabilitation program. Lynggaard et al. [37] found that patients’ education and learning of coping improves patients’ adherence to rehabilitation.

### 4.3. Feeling a Need

This domain was affected by the usefulness of the CR program and being influenced by others to attend to the CR program. Desire and motivation are prerequisites for creating a change in behavior [38]. Understanding the benefits of CR after a cardiac event encourages patients to change their lifestyle. If patients are highly motivated to get involved in their healthcare activities, their adherence to the rehabilitation program is facilitated [35]. According to Parminder et al. [39], motivation and autonomy in decision making significantly predicted persistence with the rehabilitation program indicating the need to pay attention to patients’ psychological needs and interests for designing such programs.

### 4.4. Personal Control over the Situation

Adherence to CR was influenced by patients’ income, cost of recovery, suffering from other diseases, availability of rehabilitation centers and patients’ ability to integrate the CR program sessions into the lifestyle. Social support refers to social and environmental factors that may be beyond an individual’s control [40]. Consistently, Balady et al. [41] stated that common barriers to the active attendance at CR were a lack of social support, responsibilities at home in caretaking roles, a residence far away from CR centers, and inability to take time off from work. McCarthy et al. [35] reported that suffering from other diseases, a lack of insurance coverage, or financial concerns were barriers to patients’ adherence to the program. These findings correspond with the results reported by Mair et al. [42] indicating that travel/labor dispute, personal/family problems, and low initial provision were main factors contributing to absenteeism from a CR program. According to Meng et al. [43] the patient-centered self-management program might be more effective in certain self-management outcomes than a usual care education in both short- and long-term periods.

### 4.5. Encouragement and Advice

Social factors from numerous sources, such as healthcare providers, family and neighbors, co-workers, and different types of communication influenced health behaviors. Patients’ family, friends and relatives, rehabilitation staff, other patients, timely referrals, and encouragement to attend and continue with the CR program improve patients’ attendance and adherence to the CR program [40]. Healthcare professionals can recommend participation in the CR program to all eligible patients as a part of their therapeutic regimen [44]. Communication by healthcare staff encourages the patient to adhere to the program [14,15]. The type of healthcare provider that refers a patient to a CR program also has an integral modulating role in the patient’s ultimate participation and adherence to the program [45]. According to De Gruyter et al. [46] a greater uptake of the CR program needs a direct translation of the benefits of the program for the society, incorporation of CR into standard practice, and provision of support.

### 4.6. Limitations and Suggestions for Future Studies

Insufficient numbers of patients referred to the rehabilitation centers influenced the sample size in this study. Therefore, the researchers could recruit 210 patients as the fair number of samples to examine the psychometric properties of the CRAT. Therefore, it is suggested that the CRAT is re-examined using a larger sample size and in other cultures and contexts in future studies. In addition, the predictive validity of the CRAT was not studied during its development. Additionally, this study was conducted in just two CR centers with Farsi-speaking patients using a non-random sampling method, which limited generalizability of the results. Further studies should be performed with different and greater random samples to assess the CRAT’s predictive validity for improving its application in clinical practice.

## 5. Conclusions

The CRAT, as an evaluation tool, was designed to assess patients’ reasons for adherence or non-adherence to the CR program. In comparison with available instruments, the CRAT is more comprehensive and encompasses various individual and organizational factors influencing patients’ adherence to CR program. Moreover, given performing a comprehensive literature review for item generation, the CRAT approximately covers all main items related to patients’ adherence or non-adherence to CR programs stated in previous national and international studies. The average time to complete the CRAT by a patient is about 15–20 min. Therefore, it is easily administered and is relatively quick to be completed. Also, the researchers propose that the CRAT is used in different social contexts and health care systems.

Data collected using the CRAT can be used for designing successful CR programs with the consideration of factors influencing patients’ adherences to the program. In addition, healthcare professionals can apply it to identify patients at the risk of leaving a CR program and design strategies to increase patients’ likelihood of adherence to it.

## Figures and Tables

**Table 1:** Available instruments of patients’ participation and adherence to cardiac rehabilitation.

Authors, (Year)	Methods	Focus	Cronbach’s alpha	The Studies’ Characteristics
Shanmugasegaram et al. (2012) [13]	The barriers’ scale to engage in cardiac rehabilitation and the scale of beliefs about cardiac rehabilitation.	Psychometric properties of barriers to the cardiac rehabilitation program scale (cardiac rehabilitation barriers scale).	Test-retest method with an interclass correlation coefficient 0.64.	No assessment of barriers to attendance to this program and its follow-up.
Ghisi et al. (2013a) [14]	The barriers’ scale-English version.	Barriers to the cardiac rehabilitation program in Brazil (cardiac rehabilitation barriers scale).	Cronbach’s alpha coefficient 0.85.	Significant differences in the sociodemographic and clinical characteristics, which affect barriers to cardiac rehabilitation.
Ghisi et al. (2013b) [15]	Review of literature.	Development and psychometric properties of the tool for the evaluation of the information required in cardiac rehabilitation (information needs in cardiac rehabilitation tool).	Cronbach’s alpha > 0.7.	Evaluation of patients’ needs during cardiac rehabilitation. No construct validity assessment.
Rosneck et al. (2014) [16]	Knowledge and management of cardiovascular diseases among patients in cardiac rehabilitation in step B.	Development and psychometric properties of knowledge about the disease and management tool and risk factors of cardiovascular diseases (cardiac knowledge assessment tool).	Cronbach’s alpha 0.85.	Applicable to patients in phase 2 of cardiac rehabilitation.

**Table 2:** Demographical characteristics of the subjects (*n* = 210).

Variable		*n* (%)
**Gender**	Male	122 (58.1)
	Female	88 (41.9)
**Marital status**	Single	12 (5.7)
	Married	182 (86.7)
	Widow/Widower	11 (5.2)
	Divorced	5 (2.4)
**Insurance status**	With health insurance	195 (92.9)
	Without health insurance	15 (7.1)
**Education level**	Primary school	31 (14.8)
	Secondary school	108 (51.4)
	Academic	71 (33.8)
**Job status**	Unemployed	6 (2.9)
	Housewife	70 (33.3)
	Employee	28 (13.3)
	Retired	87 (41.5)
	Self-employment	19 (9)
**Smoking and substance abuse**	Smoking	112 (54.2)
	Tobacco	25 (11.9)
	Substance abuse	3 (1.5)
	Alcohol consumption	9 (3.4)
	Smoking and substance abuse	3 (1.5)
	Smoking and alcohol consumption	8 (3.8)
	No consumption	50 (23.7)
	Mean ± SD	Range
**Age, year**	55.10 ± 1.87	39–71

**Table 3:** Items, factor loadings, Cronbach’s alpha, and observed variances *.

Dimension	Item	Factor Loading
The acceptance of the rehabilitation center % of variance = 11.71 Cronbach’s alpha: 0.814	(1) I know that cardiac rehabilitation is beneficial for my health.	0.263
	(2) My admission in the rehabilitation center was conducted harshly.	0.448
	(3) In my opinion, the environment of rehabilitation center is fun.	0.71
	(6) Staff of the rehabilitation center are experts and educated professionals.	0.678
	(7) Staff of the rehabilitation team can perfectly supervise me during the program.	0.655
	(8) The rehabilitation program provides me with adequate facilities and equipment.	0.481
	(15) Participating in the program has a positive effect on my mood.	0.572
	(18) I like other participants in the program, because we have similar problems and interests.	0.41
	(28) I was referred to the program quickly after being diagnosed with the heart disease.	0.434
	(32) Following instructions given by the cardiac rehabilitation team is the best way to stop my disease progression.	0.523
	(39) Communicating with the rehabilitation team creates a sense of ability in me.	0.422
	(41) My participation in the program makes other to feel that I am disabled.	0.403
	(43) I always pay attention to the recommendations of cardiac rehabilitation nurses.	0.584
	(50) In my opinion, the services provided in this rehabilitation center are adequate.	0.385
	(51) The schedule of classes in the rehabilitation center is not suitable for me.	0.62
	(57) The rehabilitation center always pays attention to my satisfaction.	0.364
	(59) My condition has been paid attention to in the offered program.	0.431
	(67) I believe that the heart disease cannot beat me.	0.573
	(70) I consider cardiac rehabilitation as a kind of fun.	0.451
Being interested in health % of variance = 21.93 Cronbach’s alpha: 0.723	(12) I consider my condition appropriate to continue participation in the cardiac rehabilitation program.	0.516
	(29) In my opinion, my heart condition can be improved by the program.	0.502
	(37) My family also participates in the program organized by the rehabilitation center.	0.452
	(40) My friends often encourage me to continue rehabilitation.	0.591
	(53) I am uninterested in participating in long-term programs.	0.408
	(55) Nobody notices my success in the program.	0.388
	(56) I think that relaxation training classes in the rehabilitation center is useful for me.	0.473
	(60) Recommendation of attendance to the program by the rehabilitation team increases my interest to the program.	0.55
	(63) I am sure that if I leave the program, my heart problems will return.	0.548
	(64) In my opinion, the program should be lifelong and permanent.	0.484
	(65) I have received enough information about medications through the program.	0.635
	(66) I will continue the program even if my heart condition is improved.	0.445
	(69) As required, I can have a medical visit in the rehabilitation center.	0.486
Feeling a need % of variance = 30.69 Cronbach’s alpha: 0.802	(13) I do not have enough time to participate in rehabilitation.	0.586
	(16) I do not like team sport.	0.471
	(17) I am sure that I can manage heart problems alone and I do not need cardiac rehabilitation.	0.575
	(20) I am not interested in exercise.	0.517
	(21) The physician did not tell me that I need to continue the program.	0.555
	(22) I have recovered and I do not need the program anymore.	0.545
	(24) I dislike attending training classes.	0.67
	(30) I am directly responsible for my disease problems and I do not need help from others.	0.566
	(33) I am not in the mood for adherence to a strict diet regimen.	0.63
Personal control over the situation % of variance = 39.38 Cronbach’s alpha: 0.843	(10) The cost of cardiac rehabilitation is high for me.	0.577
	(23) I cannot participate in the program because I suffer from other diseases.	0.762
	(25) In my opinion, I do not need to follow the program in an orderly and regular manner.	0.713
	(31) I do not need to continue the program because my heart problems are not serious.	0.521
	(35) I think the program can be implemented without the supervision of healthcare staff.	0.708
	(38) I prefer to follow the program in my own home rather than to go to a rehabilitation center.	0.62
	(42) The lack of coordination between the members of the cardiac rehabilitation team leads me to give up participation in the program.	0.488
	(44) The program is difficult for me.	0.51
	(47) The recommended diet regime is difficult for me in terms of performance (cost and access).	0.442
	(58) The cardiac program offered in this center is very complicated.	0.513
	(62) I am able to observe my program in all conditions.	0.073
	(68) I struggle to use drugs regularly.	0.597
Encouragement and advice % of variance = 45.22 Cronbach’s alpha: 0.612	(26) The cardiac rehabilitation team encourages me to continue rehabilitation.	0.466
	(46) I do not understand the various aspects of the program.	0.681
	(54) There is no adaptation between my culture and provided recommendations in the rehabilitation center.	0.663
	(49) It is difficult for me to access the cardiac rehabilitation center.	0.505

* The permission to use the CRAT in future studies is granted by the authors ONLY with a full citation to this article.

## Supplementary Materials

The following are available online at https://www.mdpi.com/2308-3425/6/3/25/s1, Table S1: The factor analysis of the items.

## References

[1] World Health Organization (WHO) (2019). Cardiovascular Diseases. https://www.who.int/health-topics/cardiovascular-diseases/.

[2] Rich M.W., Chyun D.A., Skolnick A.H., Alexander K.P., Forman D.E., Kitzman D.W., Tirschwell D.L. (2016). Knowledge gaps in cardiovascular care of the older adult population. A scientific statement from the American Heart Association, American College of Cardiology, and American Geriatrics Society. J. Am. Coll. Cardiol..

[3] Ezzati M., Obermeyer Z., Tzoulaki I., Mayosi B.M., Elliott P., Leon D.A. (2015). Contributions of risk factors and medical care to cardiovascular mortality trends. Nat. Rev. Cardiol..

[4] Mozaffarian D., Benjamin E.J., Go A.S., Arnett D.K., Blaha M.J., Cushman M., Howard V.J. (2016). Executive summary: Heart Disease and Stroke Statistics-2016 update: A report from the American Heart Association. Circulation.

[5] Roth G.A., Nguyen G., Forouzanfar M.H., Mokdad A.H., Naghavi M., Murray C.J. (2015). Estimates of global and regional premature cardiovascular mortality in 2025. Circulation.

[6] Heran B.S., Chen J.M., Ebrahim S., Moxham T., Oldridge N., Rees K., Taylor R.S. (2016). Exercise-based cardiac rehabilitation for coronary heart disease. Cochrane Database Syst. Rev..

[7] Gaalema D.E., Higgins S.T., Shepard D.S., Suaya J.A., Savage M.P.D., Ades P.A. (2014). State-by-state variations in cardiac rehabilitation participation are associated with educational attainment, income, and program availability. J. Cardiopulm. Rehabil. Prev..

[8] Beatty A.L., Fukuoka Y., Whooley M.A. (2013). Using mobile technology for cardiac rehabilitation: A review and framework for development and evaluation. J. Am. Heart Assoc..

[9] Shahsavari H., Shahriari M., Alimohammadi N. (2012). Motivational factors of adherence to cardiac rehabilitation. Iran J. Nurs. Midwifery Res..

[10] Murray K.A., Murphy D.J., Clements S.J., Brown A., Connolly S.B. (2014). Comparison of uptake and predictors of adherence in primary and secondary prevention of cardiovascular disease in a community-based cardiovascular prevention programme (MyAction Westminster). J. Public Health (Oxf.).

[11] De Vos C., Li X., Van Vlaenderen I., Saka O., Dendale P., Eyssen M., Paulus D. (2013). Participating or not in a cardiac rehabilitation programme: Factors influencing a patient’s decision. Eur. J. Prev. Cardiol..

[12] Karmali K.N., Davies P., Taylor F., Beswick A., Martin N., Ebrahim S. (2014). Promoting patient uptake and adherence in cardiac rehabilitation. Cochrane Database Syst. Rev..

[13] Shanmugasegaram S., Gagliese L., Oh P., Stewart D.E., Brister S.J., Chan V., Grace S.L. (2012). Psychometric validation of the cardiac rehabilitation barriers scale. Clin. Rehabil..

[14] Ghisi G.L., dos Santos R.Z., Aranha E.E., Nunes A.D., Oh P., Benetti M., Grace S.L. (2013). Perceptions of barriers to cardiac rehabilitation use in Brazil. Vasc. Health Risk Manag..

[15] Ghisi G.L., Grace S.L., Thomas S., Evans M.F., Oh P. Development and psychometric validation of a scale to assess information needs in cardiac rehabilitation: The INCR Tool. Patient Educ. Couns..

[16] Rosneck J.S., Hughes J., Gunstad J., Josephson R., Noe D.A., Waechter D. (2014). Development and psychometric evaluation of a cardiovascular risk and disease management knowledge assessment tool. J. Cardiovasc. Nurs..

[17] Bradley C. (2013). Handbook of Psychology and Diabetes: A Guide to Psychological Measurement in Diabetes Research and Practice.

[18] Polit D.F., Beck C.T., Owen S.V. (2007). Is the CVI an acceptable indicator of content validity? Appraisal and recommendations. Res. Nurs. Health.

[19] Lawshe C.H. (1975). A Qualitative Approach to Content Validity. Pers. Psychol..

[20] Martınez-Gonzalez M.A., De Irala J., Faulin F.J. (2001). Bioestadistica Amigable.

[21] Munro B.H. (2005). Statistical Methods for Health Care Research.

[22] Pett M.A., Lackey N.R., Sullivan J.J. (2003). Making Sense of Factor Analysis. The Use of Factor Analysis for Instrument Development in Health Care Research.

[23] Waltz C.F., Strickland O.L., Lenz E.R. (2010). Measurement in Nursing and Health Research.

[24] Krishnan V. (2013). The Early Child Development Instrument (EDI): An Item Analysis Using Classical Test Theory (CTT) on Alberta’s Data. https://cloudfront.ualberta.ca/-/media/ualberta/faculties-and-programs/centres-institutes/community-university-partnership/research/ecmap-reports/ediitemanalysisctt.pdf.

[25] Baumgatner T.A., Chung H. (2001). Confidence limits for intraclass reliability coefficient. Meas. Phys. Educ. Exerc. Sci..

[26] Marsh H.W., Barnes J., Cairns L., Tidman M. (1984). Self-description questionnaire: Age and sex effects in the structure and level of self-concept for preadolescent children. J. Educ. Psychol..

[27] Miller M.B. (1995). Coefficient alpha: A basic introduction from the perspectives of classical test theory and structural equation modeling. Struct. Equ. Modeling.

[28] Flodén A., Lennerling A., Fridh I., Rizell M., Forsberg A. (2011). Development and psychometric evaluation of the instrument: Attitudes towards organ donor advocacy scale (ATODAS). Open Nurs. J..

[29] Scholz U., Sniehotta F.F., Schwarzer R. (2005). Predicting physical exercise in cardiac rehabilitation: The role of phase-specific self-efficacy beliefs. J. Sport Exerc. Psychol..

[30] Schwarzer R. (2008). Modeling health behavior change: How to predict and modify the adoption and maintenance of health behaviors. J. Appl. Psychol..

[31] Turk-Adawi K.I., Oldridge N.B., Tarima S.S., Stason W.B., Shepard D.S. (2013). Cardiac rehabilitation patient and organizational factors: What keeps patients in programs?. J. Am. Heart Assoc..

[32] Resurrección D.M., Motrico E., Rubio-Valera M., Mora-Pardo J.A., Moreno-Peral P. (2018). Reasons for dropout from cardiac rehabilitation programs in women: A qualitative study. PLoS ONE.

[33] Pesah E., Supervia M., Turk-Adawi K., Grace S.L. (2017). A review of cardiac rehabilitation delivery around the world. Prog. Cardiovasc. Dis..

[34] Sniehotta F.F., Scholz U., Schwarzer R. (2006). Action plans and coping plans for physical exercise: A longitudinal intervention study in cardiac rehabilitation. Br. J. Health Psychol..

[35] McCarthy M.M., Dickson V.V., Chyun D. (2011). Barriers to cardiac rehabilitation in women with cardiovascular disease: An integrative review. J. Cardiovasc. Nurs..

[36] Reges O., Vilchinsky N., Leibowitz M., Khaskia A., Mosseri M., Kark J.D. (2013). Illness cognition as a predictor of exercise habits and participation in cardiac prevention and rehabilitation programs after acute coronary syndrome. BMC Public Health.

[37] Lynggaard V., Nielsen C.V., Zwisler A.D., Taylor R.S., May O. (2017). The patient education-Learning and Coping Strategies—Improves adherence in cardiac rehabilitation (LC-REHAB): A randomised controlled trial. Int. J. Cardiol..

[38] Ryan P. (2009). Integrated theory of health behavior change: Background and intervention development. Clin. Nurse Spec..

[39] Parminder F., Brawley L., McMahon C., Locke S., Chipperfield D. (2016). Problem-solving differences in exercise behaviour motivation in cardiac rehabilitation participants. JEMS.

[40] Kosteli M.C., Heneghan N.R., Roskell C., Williams S.E., Adab P., Dickens A.P., Greenfield S. (2017). Barriers and enablers of physical activity engagement for patients with COPD in primary care. Int. J. Chron. Obstruct. Pulmon Dis..

[41] Balady G.J., Ades P.A., Bittner V.A., Franklin B.A., Gordon N.F., Thomas R.J., Yancy C.W. (2011). Referral, enrollment, and delivery of cardiac rehabilitation/secondary prevention programs at clinical centers and beyond. Circulation.

[42] Mair V., Breda A.P., Nunes M.E.B., Matos L.D.N.J.D. (2013). Evaluating compliance to a cardiac rehabilitation program in a private general hospital. Einstein (Sao Paulo).

[43] Meng K., Musekamp G., Schuler M., Seekatz B., Glatz J., Karger G., Kiwus U., Knoglinger E., Schubmann R., Westphal R. (2016). The impact of a self-management patient education program for patients with chronic heart failure undergoing inpatient cardiac rehabilitation. Patient Educ. Couns..

[44] Buckley J.P., Furze G., Doherty P., Speck L., Connolly S., Hinton S., Jones J.L. (2013). BACPR scientific statement: British standards and core components for cardiovascular disease prevention and rehabilitation. Heart.

[45] Menezes A.R., Lavie C.J., Forman D.E., Arena R., Milani R.V., Franklin B.A. (2014). Cardiac rehabilitation in the elderly. Prog. Cardiovasc. Dis..

[46] De Gruyter E., Ford G., Stavreski B. (2016). Economic and social impact of increasing uptake of cardiac rehabilitation services—A cost benefit analysis. Heart Lung Circ..

