# Supplementary material for: Development and Psychometric Evaluation of the Cardiac Rehabilitation Adherence Tool (CRAT)
Source: J Cardiovasc Dev Dis. 2019 Jul 9;6(3):25. doi: 10.3390/jcdd6030025 (PMC6787721; doi:10.3390/jcdd6030025)
Supplement: Supplementary file 1 [file jcdd-06-00025-s001.pdf]

Table S1: The factor analysis of the items.

Item	Dimension					Item	Dimension				
	1	2	3	4	5		1	2	3	4	5
**1**	0.263	0.033	-0.089	0.0426	0.000	**38**	0.130	0.078	0.112	0.620	0.267
**2**	0.448	0.195	0.012	0.150	0.086	**39**	0.422	0.446	0.313	0.182	-0.008
**3**	0.710	-0.011	0.061	0.169	0.295	**40**	0.244	0.591	0.009	0.046	-0.011
**4**	0.464	-0.031	0.440	-0.081	0.191	**41**	0.403	0.309	0.367	0.183	0.077
**5**	0.255	-0.045	0.300	0.105	0.358	**42**	0.019	-0.074	-0.211	0.488	-0.078
**6**	0.678	0.102	0.062	0.143	0.122	**43**	0.584	0.179	0.246	0.092	0.030
**7**	0.655	0.054	0.087	0.079	0.208	**44**	0.011	0.343	0.112	0.510	0.016
**8**	0.481	0.189	0.247	0.175	0.243	**45**	0.091	0.406	0.034	0.458	-0.041
**9**	0.334	0.248	0.024	0.191	-4.759-5	**46**	0.039	0.141	0.305	0.097	0.681
**10**	0.168	0.048	0.021	0.577	0.276	**47**	0.188	0.71	0.220	0.442	0.0181
**11**	0.008	0.166	0.132	0.223	0.253	**48**	0.548	0.010	0.409	0.081	-0.091
**12**	0.257	0.516	0.363	0.220	-0.036	**49**	0.158	-0.090	0.237	0.131	0.505
**13**	0.203	0.310	0.586	0.099	0.096	**50**	0.385	0.184	-0.007	0.097	0.612
**14**	0.233	-0.145	0.350	0.280	0.382	**51**	0.620	0.051	0.555	-0.097	0.280
**15**	0.572	0.180	0.060	0.300	0.045	**52**	0.415	0.431	0.050	0.106	0.296
**16**	0.186	0.264	0.471	-0.039	0.082	**53**	0.370	0.408	0.299	-0.079	0.270
**17**	0.201	0.067	0.575	0.267	0.002	**54**	0.145	0.243	0.127	0.096	0.633
**18**	0.410	0.250	0.235	0.121	0.082	**55**	0.207	0.388	0.337	0.205	0.196
**19**	0.005	0.152	0.065	0.046	0.185	**56**	0.267	0.473	0.163	-0.013	-0.087
**20**	0.269	0.219	0.517	0.235	0.099	**57**	0.364	0.406	0.238	-0.032	0.331
**21**	0.028	-0.028	0.555	0.232	0.021	**58**	0.265	0.047	0.255	0.513	0.024
**22**	0.134	0.347	0.545	0.291	-0.033	**59**	0.043	0.376	0.359	-0.030	0.194
**23**	0.015	0.187	0.089	0.762	0.093	**60**	0.244	0.550	0.275	0.048	-0.084
**24**	0.080	0.206	0.670	0.165	0.084	**61**	0.092	0.015	0.054	0.227	0.164
**25**	0.008	-0.013	0.176	0.713	0.120	**62**	0.480	0.339	0.304	-0.073	0.266
**26**	0.225	0.102	-0.061	-0.001	0.466	**63**	0.297	0.548	0.005	0.013	0.204
**27**	0.342	0.357	0.299	0.179	-0.189	**64**	0.242	0.484	0.254	-0.062	0.117
**28**	0.434	0.304	-0.229	0.190	0.254	**65**	-0.233	0.635	0.095	-0.137	0.336
**29**	0.251	0.502	0.145	0.233	0.190	**66**	0.312	0.445	0.249	0.090	0.300
**30**	-0.100	0.099	0.566	0.038	0.183	**67**	0.573	0.151	0.208	0.200	0.091
**31**	0.022	-0.084	0.216	0.521	0.214	**68**	0.135	0.036	0.056	0.597	-0.004
**32**	0.523	0.144	0.154	0.245	0.108	**69**	-0.096	0.486	-0.048	0.086	0.153
**33**	0.395	0.052	0.630	0.084	0.210	**70**	0.451	0.359	0.221	0.058	0.279
**34**	0.122	0.074	0.091	-0.020	0.032	**71**	0.425	0.459	0.262	0.085	0.289
**35**	0.110	0.023	0.007	0.708	-0.112						
**36**	0.030	-0.013	0.119	0.281	-0.044						
**37**	0.114	0.431	-0.131	-0.042	0.452						
